# Convergent and predictive validity of the activities-specific balance confidence scales and balance recovery confidence scale, with regard to the falls efficacy scale-international: a cross-sectional study

**DOI:** 10.3389/fragi.2025.1330612

**Published:** 2025-05-30

**Authors:** Hazel Xu Teng Ting, Jiaying Ho, Peck Hoon Ong, William R. Young, Shawn Leng Hsien Soh

**Affiliations:** ^1^ Rehabilitation Services, Yishun Community Hospital, Singapore, Singapore; ^2^ Health and Social Sciences Cluster, Singapore Institute of Technology, Singapore, Singapore; ^3^ Public Health and Sport Sciences, University of Exeter, Exeter, United Kingdom

**Keywords:** falls efficacy, balance confidence, balance recovery confidence, concerns about falling, psychological constructs, psychometric, community-dwelling, older adults, fall prevention

## Abstract

The purpose of this study was to determine the convergent validity between the Activities-specific Balance Confidence (ABC) Scale, Balance Recovery Confidence (BRC) Scale and Falls Efficacy Scale-International (FES-I), which are assessment tools used to measure the constructs of falls efficacy and concerns about falling. The study also investigated the predictive validity of ABC and BRC on concerns about falling. One hundred and thirty-one older adults (mean age of 73.5 years, SD 4.98) completed the three scales and self-reported their demographic data. 63.4% were female. The convergent validity between the ABC, BRC, and FES-I scales was investigated using Pearson correlation coefficients. Predictive validity was investigated using regression models. Findings indicated strong correlation between ABC and FES-I (r = −0.794, p < 0.001), and moderate correlation between BRC and FES-I (r = −0.587, p < 0.001) and ABC and BRC (r = 0.642, p < 0.001). ABC (*R* ^2^ = 0.6279) was found to be a stronger predictor of FES-I than BRC (*R* ^2^ = 0.3398). In conclusion, assessment tools for balance confidence, balance recovery confidence, and concerns about falling should be appropriately selected when studying the various constructs of interest, instead of using them interchangeably. Concerns about falling can also be further understood by exploring balance confidence and balance recovery confidence.

## Introduction

Falls has been identified as a major health issue among older adults and is ranked as the second most common reason for unintentional injury deaths worldwide (World Helath Organization, 2021). In addition to physical and social consequences, falls also result in psychological consequences such as reduction in activity due to decreased confidence in mobility, leading to physiological and functional declines (Moore and Ellis, 2008). The 2022 World Guidelines for Falls Prevention and Management for Older Adults has recommended a holistic approach to the assessment of older adults, by including concerns about falling on top of balance and gait assessment (Montero-Odasso et al., 2022). In the literature of falls prevention practice, falls efficacy and fear of falling are key psychological factors that have been given much attention. Distinguishing falls efficacy and fear of falling is important because these two concepts are distinct even though they are closely related. Falls efficacy, rooted in Bandura’s self-efficacy theory, is a cognitive element referring to the perceived ability to prevent and manage falls (Soh, 2022a). Falls efficacy has been posited as a multidimensional construct encompassing four domains: balance confidence, balance recovery confidence, safe-landing confidence, and post fall recovery confidence (Soh, 2022a). Previously, falls efficacy has been considered to be synonymous to balance confidence (Hadjistavropoulos et al., 2011). Since then, the focus has largely been on balance confidence when targeting falls efficacy both in research and in practice (Soh et al., 2022a). However, a broader concept of falls efficacy allows different self-efficacies to be reflected and attended to when older adults need to develop their agencies to deal with falls (Soh et al., 2021a). The knowledge about the relationships between the different falls-related self-efficacies, for example, how closely balance recovery confidence is related to balance confidence, is currently limited. There is a need for a greater understanding of balance recovery confidence to advance fall prevention and management practice.

Falls efficacy has often been conflated with fear of falling (Soh, 2022a). The construct of fear of falling is generally considered to represent an emotional state that can have clear impacts on behaviour (e.g., activity avoidance) (Hughes et al., 2015). Fear of falling has been postulated to serve either as a protective or maladaptive mechanism for older adults to guard against falls (Ellmers et al., 2022). Rather than “fear of falling”, some researchers and the recent World Falls Guidelines have proposed that the term “concerns about falling” should be used instead (Ellmers et al., 2023). Concerns appear to be a less intense emotion than fear, as fear could imply analogy to phobias (Tinetti et al., 1990). Thus, older adults may find concerns about falling more relatable, more accurate in describing what they experience, and more socially acceptable to reveal (Yardley et al., 2005). Concerns about falling is prevalent in both older adults who have fallen before and those who have not yet fallen (Hadjistavropoulos et al., 2011). Having high concerns raises the risk of frailty (de Souza et al., 2022), decreased activity participation (Greenberg et al., 2016), and poorer quality of life (Schoene et al., 2019). Given the complexity of understanding the different concepts, there is a need for more evidence surrounding various falls efficacy and concerns about falling assessment tools for one to consider in their decision-making process when selecting the most appropriate tool for use (Hughes et al., 2015).

Several assessment tools, such as the Activities-specific Balance Confidence (ABC) Scale, Balance Recovery Confidence (BRC) Scale, and Falls Efficacy Scale-International (FES-I) have been developed to measure falls efficacy or concerns about falling. The 16-item ABC was constructed to assess how confident individuals were to perform various activities without losing balance or experiencing a sense of unsteadiness (Powell and Myers, 1995). Since its development in 1995, several studies have demonstrated good psychometric properties of the ABC to determine balance confidence in older adults (Soh et al., 2021b) (Stasny et al., 2011). Another tool, the newly-developed 19-item BRC, was constructed to assess how confident individuals were able to arrest falls in response to different perturbations such as a slip, a trip or a loss of balance from volitional movement (Soh, 2022b). This balance recovery confidence measure was developed in 2022 after a systematic review of falls efficacy instruments identified an absence of a suitable instrument to measure balance recovery confidence (Soh et al., 2021b). The psychometric study of BRC scale has demonstrated excellent test-retest reliability when the assessment was administered to community-dwelling older adults during a 1-week time frame (ICC_3,1_ = 0.944, 95% CI of 0.891 and 0.969) and high internal consistency (α = 0.975) (Soh, 2022b). Finally, the 16-item FES-I, is a widely used tool to assess how concerned individuals were about falling when performing different activities of daily living (Yardley et al., 2005; McGarrigle et al., 2023). This measure of concerns about falling was developed in 2005 after a series of meetings between members of the Prevention of Falls Network Europe (ProFaNE) (Yardley et al., 2005). The FES-I and its translated versions have been validated by numerous international studies to have excellent psychometric properties (Healthy Ageing Research Group).

There have been limited studies investigating the construct validity between ABC, BRC, and the FES-I scale and the predictive validity of the BRC scale on concerns about falling as the BRC scale has been recently developed. Previous studies had reported moderate-strong convergent validity between ABC and FES-I in various populations such as Iranian older adults (r = −0.48) (Taheri-Kharameh et al., 2022), Portuguese older adults (r = −0.85) (Figueiredo and Santos, 2017), Japanese post-stroke individuals (r = −0.77) (Ishige et al., 2020), and persons with balance and vestibular dysfunction (r = −0.84) (Morgan et al., 2013). Further, there are no studies reporting the relationships of these measures in the Singapore community-dwelling older adult population, despite Singapore being the second fastest ageing country (Luk, 2023). In Singapore, one in three community-dwelling older adults aged 65 years and above will have at least one fall (World Health Organization, 2007), and the ratio increases to one in two for adults aged 80 years and above (Stalenhoef et al., 1997). Falls is a leading cause of injury among older adults in Singapore, accounting for 85% of elderly patients with trauma seen at hospitals’ emergency department (Matchar et al., 2017). More psychometric studies relating to the BRC scale are also needed to provide a greater level of confidence for its use. This study aims to determine the convergent validity of the ABC, BRC and FES-I to establish the relationship between these tools measuring similar constructs, and the predictive validity of ABC and BRC scales on concerns about falling in Singapore community-dwelling older adults.

## Methods

### Design and participants

A cross-sectional study with a convenience sample of community-dwelling older adults in Singapore was conducted between February 2021 and May 2023. Ethical approval was obtained from the Singapore Institute of Technology Institutional Review Board (Ref. 2020098). Participants were recruited from word-of-mouth recommendation and poster circulation through contact networks of the study team members. Participants were included if they were ≥65 years old and living independently in the community with or without the use of a walking aid. Participants were excluded if they required any physical assistance from another person to walk within home, presented with clinical observable severe cognitive impairment, and were unable to provide written consent to participate in the study.

### Assessment tools

The Activities-Specific Balance Confidence (ABC) Scale (Powell and Myers, 1995): The ABC aims to assess individuals’ confidence in performing several progressively challenging balance and mobility tasks. Participants rated how confident he or she would be in maintaining balance if asked to perform a variety of activities, expressed as 0% = no confidence; 100% = total confidence. Confidence was ranked for 16 different activities such as getting into or out of a car, bending over to pick up a slipper, and walking in a crowded mall. The sum of the ratings (possible range = 0 to 1,600) was divided by 16 to get each participant’s ABC score. A higher score denoted a higher level of balance confidence.

The Balance Recovery Confidence (BRC) Scale (Soh et al., 2022b): The BRC aims to assess individuals’ confidence to recover balance across several progressively challenging near-fall scenarios depicting different perturbations, e.g., a slip, a trip, or from volitional movements. Participants rated how confident he or she would be to arrest a fall if faced with the different scenarios, expressed as 0 = cannot do at all; 10 = highly certain can do. Confidence was ranked for 19 different activities such as recover from a loss of balance while walking down a flight of steps without railings, recover from a trip while carrying groceries with both hands, and recover from a minor slip on a puddle of water. The sum of the ratings (possible range = 0–190) was divided by 19 to get each participant’s BRC score. A higher score denoted a higher level of balance recovery confidence.

The Falls Efficacy Scale-International (FES-I) (Yardley et al., 2005): The FES-I aims to assess individuals’ concerns about falling relating to basic and more demanding activities. Participants rated how concerned he or she would be if asked to perform a variety of activities, expressed as 1 = not at all concerned; 2 = somewhat concerned; 3 = fairly concerned; 4 = very concerned. The level of concern was ranked for 16 different activities such as cleaning the house, taking a bath or shower, going to the shop, and walking on an uneven surface. The sum of the ratings (possible range = 16–64) was obtained to get each participant’s FES-I score. A higher score denoted a higher level of concerns about falling.

A self-reported demographic characteristics form was used to record participant’s age, gender, level of education, living situation, number of medications consumed on a regular basis, functional mobility status, previous falls in the past year, and frequency of near falls in the past year.

### Statistical analysis

Statistical analyses were conducted using R version 4.3.1 (Beagle Scouts) (R Core Team, 2023). Descriptive statistics were used to summarise the characteristics of the sample.

Convergent validity refers to correlations between two measures believed to be reflecting similar constructs (Portney and Watkins, 2015). Pearson correlation coefficients were calculated to determine the association between the ABC, BRC and FES-I. The hypotheses regarding correlations between the measures constructed were:

1. The correlation between the ABC and BRC was predicted to be moderate and positive (r = 0.3–0.59).
2. The correlation between the ABC and FES-I was predicted to be strong and negative (r ≥ 0.6).
3. The correlation between the BRC and FES-I was predicted to be moderate and negative (r = −0.3 to −0.59).

Linear regression analyses were conducted to further explore the relationships between ABC, BRC and FES-I. The scores of ABC and BRC were individually correlated to FES-I score to analyse if ABC and BRC were able to explain individuals with high concerns about falling reflected on their FES-I score. Multiple regression analysis was then conducted to explore how the combination of ABC and BRC scores could explain FES-I. In both analyses, ABC and BRC scores were modeled as continuous variables, while FES-I score was inserted as a continuous outcome. These analyses provided insight into how the ABC and BRC scores (the independent variables) could predict the dependent outcomes of “concerns about falling”.

## Results

### Descriptive statistics

One hundred and thirty-one older adults with a mean age of 73.5 years (SD 4.98) participated in the study (Table 1). Over half (63.4%) were female. Just under half were educated up to secondary school level. Most participants do not stay alone, and 17.5% reported taking 4 or more medications a day. Almost all the participants could walk independently without aid. In the past year, almost three-quarter of participants did not experience a fall, whereas slightly more than three-quarter do not or rarely experience near fall.

**TABLE 1:** Characteristics of study participants. Numbers are in n (%) unless otherwise stated.

Study participants characteristics (*n* = 131)	Frequency (%) or Mean (SD)
Age (years), Mean (SD)	73.53 (4.98)
Gender	
Female	83 (63.4%)
Male	48 (36.6%)
Educational level	
No education	3 (2.3%)
Primary	12 (9.2%)
Secondary	60 (45.8%)
College/University	56 (42.7%)
Living situation	
Alone	23 (17.6%)
With spouse only	60 (45.8%)
With family/nonfamily	48 (36.7%)
Medication	
0	43 (32.8%)
1	27 (20.6%)
2	24 (18.3%)
3	14 (10.7%)
4	7 (5.3%)
5 or more	16 (12.2%)
Mobility	
Independent	124 (94.7%)
Uses walking stick	4 (3.1%)
Uses Quadstick	2 (1.5%)
Uses rollator frame	1 (0.8%)
Frequency of falls in the past year	
0	73.3%
1	18.3%
2 or more	8.4%
Frequency of near falls in the past year	
None or rare (once or twice in a year) experience	81.7%
Occasional (once or twice monthly, or quarterly) or frequent (once or twice daily, or weekly) experience	18.3%
Average ABC score (balance confidence), Mean (SD)	88.7 (13.2)
Average BRC score (balance recovery confidence), Mean (SD)	6.9 (2.2)
Average FES-I score (concerns about falling), Mean (SD)	23.7 (7.2)

### Convergent validity

The correlations between the different measures are reported in Table 2A strong negative correlation was found between the ABC and FES-I. (r = −0.794, p < 0.001), and a moderate negative correlation between the BRC and FES-I (r = −0.587, p < 0.001). Between ABC and BRC (r = 0.642, p < 0.001), there was a moderate positive correlation.

**TABLE 2:** Correlations between ABC, BRC, and FES-I scales.

Measures	ABC	BRC
ABC		0.642*
FES-I	−0.794*	−0.587*

Note *p < .001.

n = 131.

ABC, Activities-Specific Balance Confidence.

BRC, balance recovery confidence.

FES-I, Falls Efficacy Scale-International.

### Predictive validity of ABC and BRC on FES-I

Linear regression analysis of ABC and BRC on FES-I showed that both ABC and BRC contribute to FES-I, as reported in Table 3. ABC (R2 = 0.6279) was a stronger predictor of FES-I than BRC (*R* ^2^ = 0.3398). Combining ABC and BRC scores only marginally increased the *R* ^2^ to 0.6353 (Table 4).

**TABLE 3:** Linear regression analysis including ABC and BRC scores with FES-I score as dependent variable.

Instrument	Coefficient	Standard error	p-value	Adjusted *R* ^2^
ABC	−0.4345	0.02927	<0.001	0.6279
BRC	−1.969	0.2389	<0.001	0.3398

Note: *n* = 131. ABC, Activities-specific Balance Confidence Scale; BRC, Balance Recovery Confidence scale; and FES-I, Falls Efficacy Scale-International.

**TABLE 4:** Multiple regression analysis including a combination of ABC and BRC scores with FES-I score as dependent variable.

Instrument	Coefficient	Standard error	p-value	Adjusted *R* ^2^
ABC	−0.3882	0.03779	<0.001	0.6353
BRC	−0.4418	0.23158	0.0587	

Note: *n* = 131. ABC, Activities-specific Balance Confidence Scale; BRC, Balance Recovery Confidence scale; and FES-I, Falls Efficacy Scale-International.

## Discussion

The convergent and predictive validity of ABC, BRC, and FES-I have been evaluated in this study using a sample of high functioning older adults living independently in the community. Findings indicated that ABC and BRC as well as BRC and FES-I measured distinct constructs, even though the constructs may appear to be similar in nature and have often been used interchangeably. The correlations between ABC and FES-I demonstrated a higher level of congruency than between FES-I and BRC. Results also found that ABC and BRC were able to predict concerns about falling to varying degree. When compared against BRC, ABC was a stronger predictor of concerns about falling in individuals.

A mean ABC score of 88.7 in this population is generally higher than that of other populations in other studies. The mean ABC score of populations from previous studies was 86.9 in Taiwanese (Hwang et al., 2023), 85.74 in Portuguese (Figueiredo and Santos, 2017) and 70.1 in American (Cleary and Skornyakov, 2017) community dwelling older adults. The mean FES-I score of 23.7 in this study is comparable to a mean FES-I score of 23.5 in a Taiwanese study conducted in similar population (Kuo et al., 2021). However, it is generally lower than other population e.g., Turkish community-dwelling older adults (mean FES-I of 34.88) (Ulus et al., 2012) and Portuguese community-dwelling older adults (mean FES-I of 27.74) (Figueiredo and Santos, 2017). As BRC has not yet been used in other populations, no comparison of the population’s mean BRC score can be done. Future studies can look at investigating if these differences are due to cultural differences, or differences in characteristics within the population e.g., education and falls history.

This study has generated insights of the different constructs of ABC, BRC and FES-I by examining the relationship between these three scales. Previous systematic reviews on tools measuring fall-related psychological constructs have stressed that greater clarity in their psychometric properties is needed to help researchers and clinicians determine which tool is most suitable for measuring falls efficacy and concerns about falling as part of the holistic approach recommended by the World Guidelines (Montero-Odasso et al., 2022; Soh et al., 2022a; Jørstad et al., 2005). This study’s finding of ABC and FES-I demonstrating a high level of congruency was supported in previous studies conducted in community-dwelling older adults. Moore et al. (2004) as well as Figueiredo and Santos (2019) found ABC and FES-I to be highly correlated (r = −0.68 and r = −0.85 respectively) (Figueiredo and Santos, 2017; Moore et al., 2011). This suggested that the constructs measured in ABC and FES-I are more similar in nature compared to BRC. This may be because BRC relates to recovery from instability while ABC relates to maintenance of stability. While confidence of balance and balance recovery stems on the perceived ability surrounding balance control, the mechanisms to maintain balance and to recover balance when a loss of balance occur are different (Soh, 2022b). Change-in-support balance recovery strategies that involve rapid stepping and reaching movement are posited to be the only recourse in responding to large perturbations, even though they are also prevalent in relatively small perturbations (Maki et al., 2008). Given age-related physiological declines in these balance recovery strategies in older adults (Pijnappels et al., 2008; Pijnappels et al., 2010), it is imperative to account for their balance recovery confidence as many would underestimate or overestimate their risk of falling (Delbaere et al., 2010).

As falls efficacy and concerns about falling are similar in nature, researchers have been using falls efficacy and concerns about falling assessment tools interchangeably (Moore and Ellis, 2008). It is therefore important to reiterate that falls efficacy and concerns about fallings are distinct constructs (Soh et al., 2022a). Falls efficacy refers to the cognitive element and confidence in one’s perceived ability to perform activities without falling, whereas concerns about falling refer to an emotional state that results in activity avoidance (Moore and Ellis, 2008). While perceived abilities are likely to mediate concerns, other factors could also influence this relationship, e.g., the perceived safety of one’s environment and the vicarious experience of observing others suffering from falls-related injuries (Ellmers et al., 2022). For a case in point about balance recovery confidence, we postulate that near-falls experienced by individuals could potentially increase an older adult’s confidence in recovering their balance in response to different perturbations, such as trips or slips. Our previous studies have shown that near falls can be common among seniors, and compensatory stepping and reach-to-grasp strategies are frequently used to recover their balance (Soh et al., 2021c). Recognising that the perceived ability to recover from loss of balance is dissimilar to the perceived ability to perform activities steadily, researchers and clinicians should ensure using the most appropriate measurement instruments for the construct they want to study For example, balance confidence should be measured by a balance confidence-related scale, such as the ABC scale, and the BRC scale should be used to measure the balance recovery confidence for interventions, such as perturbation training.

Concerns about falling will be best measured by FES-I because of the strong evidence of its psychometric properties. However, clinicians will also need to work with their patients regarding potential factors such as balance or balance recovery confidence that could give rise to the concerns about falling. Without addressing the root causes, patients are likely to avoid or excessively limit the activities (Greenberg et al., 2016; Lach, 2002) as a strategy to overcome their concerns about falling. To measure balance confidence, ABC scale is of choice because of the strong evidence of its psychometric properties (Powell and Myers, 1995). BRC scale will be suitably used to measure balance recovery confidence as there are no other scales measuring this construct ((Soh et al., 2022b)). Based on the findings, ABC explained 62.8% of variance for concerns about falling, whereas BRC explained 34.0% of variance. When the combination of ABC and BRC scores was compared to FES-I score, ABC and BRC together explained 63.5% of FES-I score. Previous studies have also found that strategies adopted by an individual to maintain balance when engaging in activities can affect the development of concerns about falling (Ellmers et al., 2022). Balance confidence and balance recovery confidence could thus be useful psychological factors to inform clinicians how they could intervene behavioural responses to concerns about falling and measure the outcome of interventions. Literature has also shown that balance training exercises can improve balance confidence as reflected in improvements in ABC score (Myers et al., 1998). As BRC is a relatively new assessment tool, future studies can explore how exercises or other clinical interventions can improve balance recovery.

There are some limitations of this study. First, study participants are high functioning level older adults living in the community. The findings may not be representative of other populations such as frail older adults and people with stroke or Parkinson’s. Second, this study has only used falls efficacy tools targeting balance confidence and balance recovery confidence. The tools to measure other domains of falls efficacy such as safe falling confidence and post fall recovery confidence have not been assessed. However, we view that the convergent and predictive validity of these falls efficacy tools compared against ABC, BRC and FES-I should provide similar findings considering the nature of the constructs targeted.

In conclusion, this study has highlighted some key points advocating a proper selection of the commonly used tools measuring falls efficacy and concerns about falling. First, ABC, BRC, and FES-I are measuring similar, but distinct constructs. The conceptual nature of the constructs measured by the different instruments are reportedly different and this is demonstrated by moderate-strong levels of correlation found between the instruments. Second, ABC and BRC scores contribute to FES-I scores, suggesting that balance confidence and balance recovery confidence can be examined when targeting concerns about falling. This would provide a deeper understanding of the interventional mechanisms in a way whether concerns about falling was addressed due to changes in perceived balance control abilities or influences in other potential variables, such as a greater perceptual control towards reducing falls-related consequences (Soh, 2022a).

## Data Availability

The data supporting the findings of this study are available from the corresponding author upon reasonable request.
